# Retrograde endoscopic ultrasound-guided entero-enterostomy for the management of a high-output enterocutaneous fistula and ileal stricture in a complex surgical abdomen

**DOI:** 10.1055/a-2428-0026

**Published:** 2024-11-08

**Authors:** Sunil Gupta, Eimear Kirby, Sarang Gupta, Katarzyna M. Pawlak, Joao De Rezende-Neto, Jeffrey D. Mosko, Natalia C. Calo

**Affiliations:** 1 10071 Division of Gastroenterology, St Michaelʼs Hospital, Toronto, Canada; 2 8539 Department of Gastroenterology and Hepatology, Westmead Hospital, Sydney, Australia; 3 10071 Department of Surgery, St Michaelʼs Hospital, Toronto, Canada

A 26-year-old man sustained significant traumatic thoracoabdominal injuries following a gunshot. After multiple laparotomies, small-bowel resections, and an extended left hemicolectomy with end-colostomy formation, he developed a high-output enterocutaneous fistula (ECF) and loss of colostomy output. Computed tomography imaging confirmed an ECF from the ileum to the anterior abdominal wall. There was also a long ileal stricture distal to the fistula. Owing to his complex surgical abdomen and the proximity of the ECF to an abdominal flap, surgical reintervention was deemed high risk. He was therefore referred for endoscopic management ( Video 1 ).

A retrograde endoscopic ultrasound-guided entero-enterostomy is created in a patient with a high-output enterocutaneous fistula who had undergone multiple abdominal surgical procedures following a gunshot wound.Video 1

**Fig. 1:**
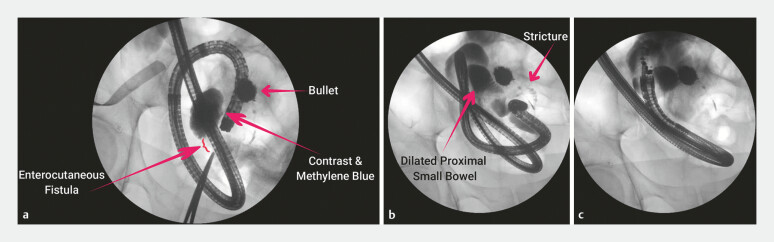
Fluoroscopic images showing: **a** filling of a dilated loop of small bowel by contrast injected from the skin side of the enterocutaneous fistula; **b, c** a deep ileal stricture and proximal dilated loops of small bowel, which were identified as a suitable target for endoscopic ultrasound-guided entero-enterostomy.

Methylene blue and contrast dye were injected from the skin side of the ECF, filling a dilated loop of small bowel. No downstream passage of contrast was noted ( Fig. 1 **a** ). Retrograde ileoscopy using a pediatric colonoscope revealed a non-traversable benign-appearing ileal stricture, 90 cm proximal to the ileocecal valve (ICV). Contrast injection demonstrated a 10-cm tortuous stricture ( Fig. 1 **b** ), extending to the previously contrast-filled loop of small bowel. Given the length and character of the stricture, endoscopic balloon dilation and enteral stenting were not feasible.

**Fig. 2:**
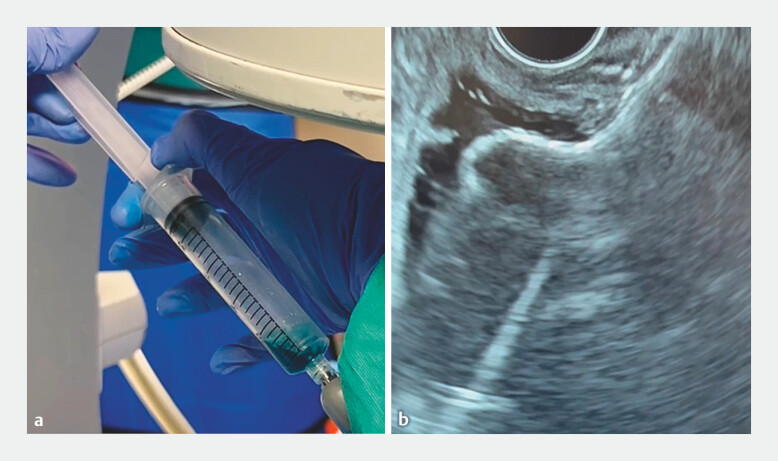
Images during creation of the endoscopic ultrasound-guided entero-enterostomy showing: **a** aspiration of methylene blue dye confirming puncture of the appropriate bowel segment; **b** deployment of the lumen-apposing metal stent.

**Fig. 3:**
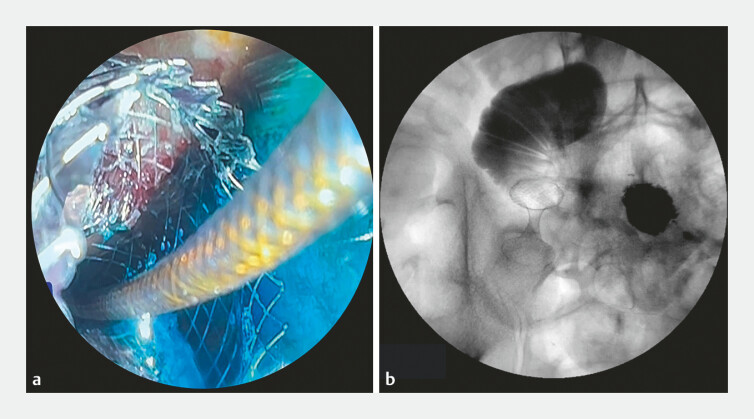
Confirmation of correct deployment of the lumen-apposing metal stent is shown: **a** on endoscopic view, by passage of methylene blue though the stent; **b** on radiographic view, by passage of contrast.

**Fig. 4:**
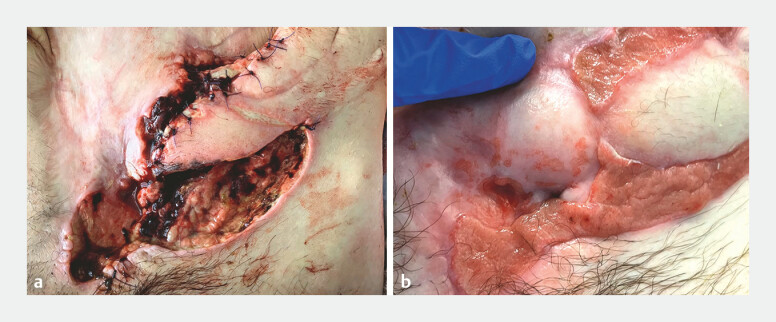
Photographs showing the abdominal flap: **a** before creation of the entero-enterostomy; **b** after entero-enterostomy formation.

We then proceeded to retrograde endoscopic ultrasound (EUS)-guided entero-enterostomy creation. With the aid of a guidewire, and under endoscopic, fluoroscopic, and endosonographic guidance, a linear echoendoscope was advanced into the ileum via the end-colostomy, cecum, and ICV. At 50 cm from the ICV, we identified an adjacent dilated loop of small bowel ( Fig. 1 **b, c** ). Water was instilled through the ECF, with the endosonographic view demonstrating filling, thereby indicating this to be upstream from the ECF. Puncture was performed with a 19-gauge needle, with subsequent aspiration of methylene blue ( Fig. 2 **a** ). We then created an EUS-guided entero-enterostomy with an electrocautery-enhanced 15-mm lumen-apposing metal stent (LAMS; Hot-AXIOS; Boston Scientific, USA) ( Fig. 2 **b** ). Passage of methylene blue and contrast through the stent confirmed its accurate deployment ( Fig. 3 ). With the ECF and stricture bypassed, the patient’s colostomy output returned, the ECF resolved, and the abdominal flap healed ( Fig. 4 ).

Although electrocautery-enhanced lumen apposition with metal stenting is well established, herein we have demonstrated a novel application of this technique in the management of a complex postsurgical trauma patient with a high-output ECF and a deep ileal stricture.

Endoscopy_UCTN_Code_TTT_1AO_2AO

